# P-1060. Social and Demographic Factors Associated with Hospital-Acquired Infections at a Large, Urban Academic Medical Center in New York City

**DOI:** 10.1093/ofid/ofaf695.1255

**Published:** 2026-01-11

**Authors:** Johain R Ounadjela, Courtney Takats, Sarah E Hochman, Madeline DiLorenzo, Ranekka Dean, Omar El Shahawy

**Affiliations:** NYU Grossman School of Medicine, New York, NY; NYU Langone Health, New York, New York; NYU Langone Health, New York, New York; NYU Langone Health, New York, New York; NYU Langone Health, New York, New York; New York University Grossman School of Medicine, New York, New York

## Abstract

**Background:**

Hospital-acquired infections (HAI) are associated with high morbidity and mortality. While studies have shown that racial and ethnic minorities have a higher risk of HAIs, few have investigated the role of other social determinants of health (SDOH) such as primary language, housing status and substance use. This study aimed to identify which SDOH are associated with a higher risk of HAI.

**Methods:**

We extracted clinical and demographic data from the electronic medical record on 2,061 patients with HAI (including catheter associated bloodstream and urinary tract infections, Clostridium difficile, and infections due to methicillin resistant Staphylococcus aureus or carbapenem resistant Enterobacterales) reported to the National Healthcare Safety Network from three urban hospitals in our health system between 2016-2023. Patients less than 18 years old or those with missing SDOH data were excluded. HAI cases were matched to controls in a 1:3 ratio based on age, sex, Charlson Comorbidity Score, level of care, and temporality. We compared cases to controls regarding race/ethnicity, preferred language, housing status, and substance use using chi-squared analyses.

**Results:**

The final cohort included 5,181 patients (Table 1). Patients with HAI were more likely to be a racial/ethnic minority (48.5% vs 37.7%; p-value: < 0.0001), have heavy alcohol use (4.9% vs 2.4%; p-value: <0.0001), have severe illicit drug use (2.8% vs 1.7%; p-value: 0.0127), have unstable housing (5.8% vs 4.0%; p-value: 0.0242, and speak a primary language other than English (18.7% vs 10.8%; p-value: < 0.0001) compared to controls. Tobacco use was not significantly associated with HAI. Patients with HAI also had more hospital admissions in the year after their initial admission (3.7 vs 0.7; p-value: < 0.0001) and longer hospital stays (29.9 vs 9.7 days; p-value: < 0.0001) compared to controls.

**Conclusion:**

In this cohort, minority race/ethnicity, heavy alcohol use, severe illicit drug use, unstable housing, and non-English primary language were significantly associated with HAIs. Future interventions should address these SDOH to reduce HAI incidence and associated morbidity and mortality.

**Disclosures:**

All Authors: No reported disclosures

